# Weight changes from young to middle adulthood in relation to blood pressure and hypertension

**DOI:** 10.7189/jogh.15.04297

**Published:** 2025-10-24

**Authors:** Xiaohui Yu, Xianwei Li, Chunxiao Xu, Xi Duan, Danru Liu, Jing Dong, Jie Ren, Junli Tang, Aiqiang Xu, Xiaolei Guo

**Affiliations:** Shandong Center for Disease Control and Prevention, Jinan, China

## Abstract

**Background:**

To address the limited evidence in Asian populations, we aimed to elucidate the association of weight changes from young to middle adulthood with blood pressure and hypertension among Chinese adults.

**Methods:**

We used data from the China Health Evaluation and Risk Reduction Through Nationwide Teamwork (ChinaHEART) project conducted in Shandong Province, China between December 2015 and November 2022. Within the ChinaHEART project, participants aged 35 to 64 years were selected, and standardised measurements (including questionnaires, physical examinations, and laboratory measurements) were performed. We used multivariable adjusted restricted cubic splines, linear regression models and logistic regression models for analysis.

**Results:**

We included 56 459 participants for analysis. Compared to stable normal weight, all other weight trajectories (maximum overweight, obesity to non-obesity, non-obesity to obesity, and stable obesity) showed positive associations with systolic and diastolic blood pressure. Hypertension risk increased progressively across these groups, with adjusted odds ratios (OR) of 1.83 (95% confidence interval (CI) = 1.73, 1.94), 2.29 (95% CI = 1.81, 2.89), 3.88 (95% CI = 3.62, 4.17), and 4.96 (95% CI = 4.09, 6.00), respectively.

**Conclusions:**

Weight gain from young to middle adulthood independently predicts elevated blood pressure and hypertension. Public health strategies should prioritise weight management across the life course to mitigate hypertension burden.

Cardiovascular disease (CVD) is the largest contributor to mortality globally [1] and accounts for 40% of total deaths in China [2]. Hypertension, a key modifiable risk factor for CVD [3], has emerged as a significant global public health concern. Data from the 2018 National Chronic Disease and Risk Factor Surveillance in China indicate that the prevalence rate of hypertension among Chinese adults is 27.5%, meaning that the condition affects approximately one in four adults [4], with the number of hypertension patients estimated to be about 245 million [5]. Preventing hypertension is, therefore, a priority for healthcare in China.

Obesity likewise remains a major public health issue in countries worldwide. Over one billion people worldwide were classified as obese in 2022, and this figure continues to grow each year [6]. Data from the China Chronic Disease and Nutrition Surveillance (2015–19) indicated that more than half of Chinese adults were either overweight (34.3%) or obese (16.4%), with the country now having the largest overweight or obese population globally [7]. Obesity is associated with a range of metabolic disorders and serves as a significant risk factor for type 2 diabetes [8] and CVD [9], as well as an independent risk factor for hypertension [10].

Multiple studies have demonstrated a substantial impact of body weight changes on hypertension risk and blood pressure levels [11–17]. In 2015, the Health in Pomerania study [11] found that a one-kilogram weight change was positively associated with a 0.45-mm Hg change in systolic blood pressure (SBP) and a 0.32-mm Hg change in diastolic blood pressure (DBP) over a five-year follow-up period. Similarly, Juhaeri and colleagues [14] found that weight gain was associated with elevated blood pressure and an increased incidence of hypertension, a finding corroborated by a large Japanese study involving 3431 men and 2409 women aged 30–69 years [15]. Clinical trials have likewise indicated that weight loss effectively reduces blood pressure [10,18,19].

Although a position paper from the Obesity Society and the American Society of Hypertension highlighted the established association between obesity and hypertension [20], different studies reported variable effect sizes for weight change on hypertension risk. These variations could be related to the different structures of their samples, with few studies examining how long-term weight trajectories influence hypertension risk in Asian populations. This presents a significant gap in knowledge, as weight typically accumulates dynamically over decades, especially during the transition from young to middle adulthood [21]. Weight trajectory patterns may thus better reflect cumulative metabolic stress than single-time weight measurements [22].

Our study addresses this gap by quantifying dose-response relationships of weight gain in an understudied Asian context. Utilising data from the China Health Evaluation and Risk Reduction Through Nationwide Teamwork (ChinaHEART) project in Shandong Province, China, we wanted to assess the association between weight changes from young to middle adulthood and blood pressure levels and hypertension risk. Given China's current heightened focus on weight management, understanding these relationships is paramount for developing effective long-term strategies and evidence-based public health interventions.

## METHODS

### Study sample

We conducted this study in Shandong Province, China, from December 2015 to November 2022. Details of the overarching ChinaHEART project have been previously described [23]. In brief, twelve representative counties/districts were selected within the project based on geographical location, economic level, and population size and structure. Individuals aged 35–75 years who were enrolled in the screening programme were invited for face-to-face interviews, where they were given questionnaires and underwent physical examination and laboratory measurement. Those with project ID numbers ending with 1, 3, 5, or 7 were randomly sampled and interviewed about their weight at the age of 25 years, as well as asked for detailed information about their lifestyle and medical history.

This study enrolled 99 165 individuals in total. After excluding 31 289 participants aged <35 or ≥65 years (n = 31 289), those who did not recall their weight at the age of 25 years (n = 11 402), and those who did not measure weight or height at middle adulthood (n = 15), we retained 56 459 respondents for our analysis (Figure S1 in the **Online Supplementary Document**). The central ethics committee at the China National Center for Cardiovascular Disease approved the ChinaHEART project, while written informed consent was obtained from all enrolled participants.

### Blood pressure measurement and definition of hypertension

Blood pressure was measured in the clinics within the selected project regions using an electronic blood pressure monitor and a standardised procedure. After a five-minute break, an interviewer measured the right upper arm blood pressure twice with a one-minute interval. If the difference between two SBP readings exceeded 10 mm Hg, a third measurement was taken, and the average of the last two measurements was used.

According to the US Joint National Committee and Chinese definitions [24–26], we defined hypertension as the mean of SBP≥140 mm Hg and/or DBP≥90 mm Hg, or self-reported diagnosis of hypertensive patients, or taking anti-hypertensive drug in the past two weeks.

### Assessments of weight change

Trained technicians measured the participants’ height and weight, recording them to the nearest 0.1 cm and 0.1 kg, respectively. Additionally, participants were asked to recall their weight at the age of 25 years. Body mass indices (BMIs) at the two time points were calculated as the corresponding weight in kilograms divided by height in meters squared.

According to the recommendations of the Working Group on Obesity in China [27], we further categorised BMI into normal weight (<24.0 kg/m^2^), overweight (24.0–27.9 kg/m^2^), and obesity (≥28.0 kg/m^2^). We established five weight change patterns based on BMI at age 25 to BMI at the survey: stable normal pattern (BMI<23.9 kg/m^2^ at both times), maximum overweight pattern (23.9–27.9 kg/m^2^ at either time, but not ≥28 kg/m^2^ at the other time), obese to non-obese pattern (≥28 kg/m^2^ at a younger age and <28 kg/m^2^ later), non-obese to obese pattern (<28 kg/m^2^ at younger age and ≥28 kg/m^2^ later), and stable obesity (≥28 kg/m^2^ at both times) [28].

We likewise categorised weight gain from young to middle adulthood into five groups: weight loss group (weight loss ≥2.5kg), stable weight group (weight change <2.5kg), small to moderate weight gain group (between ≥2.5 and <10.0 kg), moderate to large weight gain group (between ≥10.0 and <20.0 kg), and extreme weight gain group (weight gain ≥20.0 kg) [28,29].

### Covariates

Covariates included age (year), gender (male and female), ethnicity (Han and non-Han), Hukou status (urban, rural, unified residential status, and unknown), marital status (married and not married), education level (primary school or lower, middle school, high school, college or above, and unknown), occupation (farmer, nonfarmer, and unknown), household income (yuan per year: <10 000, 10 000–50 000, >50 000, and unknown), smoking status (yes and no), drinking status (yes and no), leisure time physical activity (never, 1–3 times a month, 1–2 times a week, 3–5 times a week, every day, and unknown), family history of hypertension (yes and no), and comorbidities (diabetes (with and without), CVD (with and without), chronic obstructive pulmonary disease (COPD, with and without), and cancer (with and without)).

### Statistical analysis

We calculated descriptive statistics across different weight change patterns, presenting continuous variables as means and standard deviations (SDs), and categorical variables as frequencies and percentages, and comparing different weight change patterns for the former using one-way ANOVA and the latter using χ^2^ tests.

We employed linear regression and multiple logistic regression models to assess the associations of weight change patterns with hypertension and blood pressure levels, respectively, with the stable normal weight model acting as the reference. The models were adjusted for age, gender, ethnicity, Hukou status, marital status, education level, occupation, household income, smoking status, drinking status, leisure time physical activity, family history of hypertension, and comorbidities (diabetes, CVD, COPD, and cancer). We further performed stratified analysis by gender and age groups.

We also investigated the associations between absolute weight change groups and hypertension risk and blood pressure levels. To test the robustness of our results, we treated absolute weight changes as continuous variables, while using the stable weight group as a reference for the absolute weight change groups. Restricted cubic splines with three knots (25th, 50th, and 75th centiles) were applied to flexibly model the potential dose-response relationships between weight change from young to middle adulthood and hypertension risk and blood pressure levels, while avoiding overfitting. We adjusted these analyses for all covariates in the full multivariable model. Finally, we ran sensitivity analyses to compare characteristics between the study sample and participants without recalled weight at age 25.

We used Stata, version 15.0 (StataCorp LLC, College Station, Texas, USA) and *R*, version 4.5.0 (R Core Team, Vienna, Austria) for all the statistical analyses. A two-sided *P* ≤ 0.05 indicated statistical significance.

## RESULTS

### Participant characteristics

The average age among the 56 459 participants was 53.34 years (SD = 7.38), with 61.04% being female (**Table 1**). The mean BMI was 22.21 kg/m^2^ at age 25, and 25.97 kg/m^2^ at the time of the survey. Overall, from age 25 years to their middle age, 26.75% of participants kept stable normal weight, 45.06% were in the maximum overweight group, 1.98% were in the stable obesity group, 25.05% transitioned from non-obesity to obesity with an average weight gain of 19.53 kg, while only 1.15% transitioned from obesity to non-obesity, having lost an average of 9.84 kg. Overall, participants gained 9.90 kg in weight from the age of 25 years to the time of the survey.

**Table 1:** Participant characteristics at screening by weight change patterns*

	Total (n = 56 459)	Stable normal (n = 15 103, 26.75%)	Maximum overweight (n = 25 443, 45.06%)	Obesity to non-obesity (n = 652, 1.15%)	Non-obesity to obesity (n = 14 141, 25.05%)	Stable obesity (n = 1120, 1.98%)	*P*-value†
**Age in years, x̄ (SD)**	53.34 (7.38)	52.64 (7.90)	53.60 (7.20)	56.20 (6.55)	53.47 (7.07)	53.50 (7.64)	<0.001
**Gender**							<0.001
Male	21 996 (38.96)	6026 (39.90)	10 073 (39.59)	271 (41.56)	5167 (36.54)	459 (40.98)	
Female	34 463 (61.04)	9077 (60.10)	15 370 (60.41)	381 (58.44)	8974 (63.46)	661 (59.02)	
**Ethnicity**							0.099
Han	56 311 (99.74)	15 072 (99.79)	25 381 (99.76)	650 (99.69)	14 090 (99.64)	1118 (99.82)	
Non-Han	148 (0.26)	31 (0.21)	62 (0.24)	2 (0.31)	51 (0.36)	2 (0.18)	
**Hukou status**							<0.001
Urban	48 706 (86.27)	12 997 (86.06)	21 926 (86.18)	559 (85.74)	12 242 (86.57)	982 (87.68)	
Rural	3780 (6.70)	1240 (8.21)	1653 (6.50)	52 (7.98)	772 (5.46)	63 (5.63)	
Unified residential status	3971 (7.03)	866 (5.73)	1863 (7.32)	41 (6.29)	1126 (7.96)	75 (6.70)	
Unknown‡	2 (0.00)	-	1 (0.00)	-	1 (0.01)	-	
**Marital status**							0.011
Married	54 024 (95.69)	14 496 (95.98)	24 366 (95.77)	615 (94.33)	13 471 (95.26)	1076 (96.07)	
Not married	2435 (4.31)	607 (4.02)	1077 (4.23)	37 (5.67)	670 (4.74)	44 (3.93)	
**Education level**							<0.001
Primary school or lower	26 338 (46.65)	6764 (44.79)	11 953 (46.98)	364 (55.83)	6647 (47.01)	610 (54.46)	
Middle school	22 008 (38.98)	5953 (39.42)	9828 (38.63)	202 (30.98)	5638 (39.87)	387 (34.55)	
High school	6425 (11.38)	1838 (12.17)	2941 (11.56)	72 (11.04)	1479 (10.46)	95 (8.48)	
College or above	1 291 (2.29)	484 (3.20)	540 (2.12)	9 (1.38)	234 (1.65)	24 (2.14)	
Unknown‡	397 (0.70)	64 (0.42)	181 (0.71)	5 (0.77)	143 (1.01)	4 (0.36)	
**Occupation**							<0.001
Farmer	44 153 (78.20)	11 682 (77.35)	19 915 (78.27)	517 (79.29)	11 139 (78.77)	900 (80.36)	
Non-farmer	11 923 (21.12)	3356 (22.22)	5350 (21.03)	130 (19.94)	2871 (20.30)	216 (19.29)	
Unknown‡	383 (0.68)	65 (0.43)	178 (0.70)	5 (0.77)	131 (0.93)	4 (0.36)	
**Household income in ¥/y**							<0.001
<10 000	10 724 (18.99)	2742 (18.16)	4847 (4 847)	155 (23.77)	2738 (19.36)	242 (21.61)	
10 000–50 000	37 245 (65.97)	10 066 (66.65)	16 790 (65.99)	417 (63.96)	9228 (65.26)	744 (66.43)	
>50 000	6452 (11.43)	1840 (12.18)	2827 (11.11)	51 (7.82)	1629 (11.52)	105 (9.38)	
Unknown‡	2038 (3.61)	455 (3.01)	979 (3.85)	29 (4.45)	546 (3.86)	29 (2.59)	
**Smoking status**	10 040 (17.78)	3230 (21.39)	4 417 (17.36)	121 (18.56)	2066 (14.61)	206 (18.39)	<0.001
**Drinking status**	8133 (14.41)	2288 (15.15)	3655 (14.37)	100 (15.34)	1906 (13.48)	184 (16.43)	<0.001
**Leisure time physical activity**							<0.001
Never	41 058 (72.72)	11 375 (75.32)	18 325 (72.02)	432 (66.26)	10 127 (71.61)	799 (71.34)	
1–3 times a month	1706 (3.02)	450 (2.98)	811 (3.19)	9 (1.38)	415 (2.93)	21 (1.88)	
1–2 times a week	2667 (4.72)	662 (4.38)	1217 (4.78)	37 (5.67)	693 (4.90)	58 (5.18)	
3–5 times a week	3344 (5.92)	783 (5.18)	1543 (6.06)	54 (8.28)	894 (6.32)	70 (6.25)	
Everyday	7647 (13.54)	1828 (12.10)	3526 (13.86)	120 (18.40)	2001 (14.15)	172 (15.36)	
Unknown*	37 (0.07)	5 (0.03)	21 (0.08)		11 (0.08)		
**Family history of hypertension**	2832 (5.02)	633 (4.19)	1255 (4.93)	32 (4.91)	838 (5.93)	47 (6.61)	<0.001
**Comorbidities**							
Diabetes	4189 (7.42)	650 (4.30)	1889 (7.42)	191 (29.29)	1285 (9.09)	174 (15.54)	<0.001
							
CVD	2058 (3.65)	456 (3.02)	941 (3.70)	64 (9.82)	534 (3.78)	63 (5.63)	<0.001
COPD	61 (0.17)	23 (0.23)	20 (0.12)	1 (0.23)	17 (0.20)		0.184
Cancer	100 (0.31)	25 (0.28)	57 (0.39)	1 (0.26)	15 (0.20)	2 (0.29)	0.168
**BMI in kg/m^2^, x̄ (SD)**							
At age of 25 y	22.21 (2.90)	20.59 (1.92)	22.26 (2.54)	29.49 (1.62)	22.89 (2.62)	30.07 (2.11)	<0.001
At survey	25.97 (3.58)	21.99 (1.50)	25.62 (1.45)	25.57 (1.93)	30.39 (2.18)	31.79 (3.21)	<0.001
**Absolute weight change, x̄ (SD)**	9.90 (10.05)	3.77 (5.92)	8.95 (8.26)	−9.84 (6.30)	19.53 (8.92)	4.16 (8.43)	<0.001
**Blood pressure (mmHg), x̄ (SD)**							
SBP	143.28 (20.85)	136.93 (20.28)	143.24 (20.20)	146.58 (20.59)	149.31 (20.56)	151.72 (20.69)	<0.001
DBP	85.01 (11.38)	81.50 (10.88)	84.95 (11.01)	85.18 (11.53)	88.47 (11.33)	89.88 (11.61)	<0.001
Hypertension	31 223 (55.30)	6008 (39.78)	14 029 (55.14)	428 (65.64)	9916 (70.12)	842 (75.18)	<0.001

BMI – body mass index, COPD – chronic obstructive pulmonary disease, CVD – cardiovascular diseases, DBP – diastolic blood pressure, SBP – systolic blood pressure, SD – standard deviation, x̄ – mean

*Data presented as n (%) unless specified otherwise.

†One-way ANOVA for continuous and χ^2^ test for categorical variables.

‡Participants either refused to answer the question or did not know the answer.

### Associations of weight status with blood pressure

When stratified by BMI categories at the age of 25 years in the fully adjusted model, underweight was negatively associated with SBP (correlation coefficient (*β*) = −1.88; 95% confidence interval (CI) = −2.72, −1.03; *P* < 0.001), while overweight (*β* = 2.91; 95% CI = 2.38, 3.44; *P* < 0.001) and obesity (*β* = 6.38; 95% CI = 5.18, 7.57; *P* < 0.001) were positively associated with SBP. We arrived at similar findings for BMI at age 25 years and DBP (underweight: *β* = −1.12, 95% CI = −1.61, −0.64; *P* < 0.001; overweight: *β* = 1.37, 95% CI = 1.07, 1.67; *P* < 0.001; obesity: *β* = 3.06; 95% CI = 2.39, 3.74; *P* < 0.001). Furthermore, individuals who were overweight (OR = 1.37; 95% CI = 1.29; 1.46, *P* < 0.001) and obese (OR = 2.14; 95% CI = 1.85, 2.48; *P* < 0.001) had a higher risk of hypertension, whereas being underweight was negatively associated with hypertension (OR = 0.79; 95% CI = 0.72, 0.87; *P* < 0.001). We noted similar results when we assessed the associations between BMI at survey and blood pressure (Table S1 in the **Online Supplementary Document**).

### Associations of weight change patterns and blood pressure

In the fully adjusted model that accounted for individual risk factors (**Table 2**), being maximally overweight (*β* = 5.46; 95% CI = 4.96, 5.96; *P* < 0.001), transitioning from obesity to non-obesity (*β* = 7.52, 95% CI = 5.58, 9.46; *P* < 0.001) or non-obesity to obesity (95% CI = 11.09, 12.26; *P* < 0.001), and being in the stable obesity group (*β* = 13.57, 95% CI = 12.09, 15.05; *P* < 0.001) was positively associated with SBP. We found similar relationships in the effect of weight change patterns on DBP (maximum overweight: *β* = 3.27; 95% CI = 2.99, 3.55; *P* < 0.001; obesity to non-obesity: *β* = 3.32; 95% CI = 2.23, 4.42; *P* < 0.001; non-obesity to obesity: *β* = 7.12, 95% CI = 6.79, 7.45; *P* < 0.001; stable obesity: *β* = 7.80; 95% CI = 6.97, 8.64; *P* < 0.001).

**Table 2:** Association of blood pressure with weight change patterns from young to middle adulthood*

	SBP		DBP		Hypertension	
**Weight change patterns†**	***β* (95% CI)**	***P*-value**	***β* (95% CI)**	***P*-value**	**OR (95% CI)**	***P*-value**
Stable normal (reference)	1.00		1.00		1.00	
Maximum overweight	5.46 (4.96, 5.96)	<0.001	3.27 (2.99, 3.55)	<0.001	1.83 (1.73, 1.94)	<0.001
Obesity to non-obesity	7.52 (5.58, 9.46)	<0.001	3.32 (2.23, 4.42)	<0.001	2.29 (1.81, 2.89)	<0.001
Non-obesity to obesity	11.68 (11.09, 12.26)	<0.001	7.12 (6.79, 7.45)	<0.001	3.88 (3.62, 4.17)	<0.001
Stable obesity	13.57 (12.09, 15.05)	<0.001	7.80 (6.97, 8.64)	<0.001	4.96 (4.09, 6.00)	<0.001

*β* – correlation coefficient, CI – confidence interval, OR – odds ratio

*Model was adjusted for age, gender, ethnicity, Hukou status, marital status, education level, occupation, household income, smoking status, drinking status, leisure time physical activity, and comorbidities.

†Stable normal pattern (<23.9 at both times), maximum overweight pattern (23.9–27.9 at either time, but not ≥28.0 at the other time), obesity to non-obesity pattern (≥28.0 at younger age and <28.0 later), non-obesity to obesity pattern (<28.0 at younger age and ≥28.0 later), and stable obesity (≥28.0 at both times).

We also assessed the association between weight change patterns and hypertension (**Table 2**). In the fully adjusted model, participants who gained more weight at any time from young to middle adulthood had higher ORs of hypertension of 1.83 (95% CI = 1.73, 1.94; *P* < 0.001), 2.29 (95% CI = 1.81, 2.89; *P* < 0.001), 3.88 (95% CI = 3.62, 4.17; *P* < 0.001), and 4.96 (95% CI = 4.09, 6.00; *P* < 0.001) in the maximum overweight, obesity to non-obesity, non-obesity to obesity, and stable obesity pattern groups compared with stable normal pattern group, respectively.

**Figure 1:**
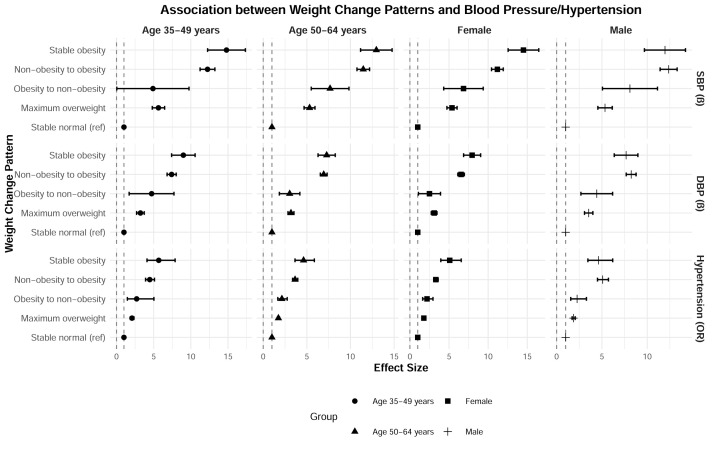
Association between weight change patterns from young to middle adulthood with blood pressure/hypertension, stratified by gender and age. Adjusted for age, gender, ethnicity, Hukou status, marital status, education level, occupation, household income, smoking status, drinking status, leisure time physical activity, family history of hypertension, and comorbidities. The solid points represent effect sizes (*β* for continuous outcomes or ORs for binary outcomes). Horizontal lines indicate 95% CIs, which reflect the precision of the effect size estimates.

Across both genders and age groups (35–49 years and 50–64 years), all weight change patterns (maximum overweight, obesity to non-obesity, non-obesity to obesity, and stable obesity) were significantly associated with higher SBP and DBP and an increased risk of hypertension compared to the stable normal weight pattern (all *P* < 0.05, with most *P* < 0.001). Notably, the non-obesity to obesity and stable obesity patterns generally showed the strongest associations, with the largest *β* coefficients for blood pressure and the highest ORs for hypertension, though specific patterns varied slightly by gender and age (**Figure 1**). However, due to small numbers in some cells and multiple comparisons without correction, these stratified results should be interpreted with caution.

### Sensitivity analyses and dose-response relationship

Compared to being in the stable weight group, being in the weight loss group was not significantly associated with blood pressure outcomes, while all weight gain groups exhibited significantly elevated SBP, DBP, and hypertension risk, even after adjusting for sociodemographic, lifestyle, and comorbidities factors (Table S2 in the **Online Supplementary Document**).

**Figure 2:**
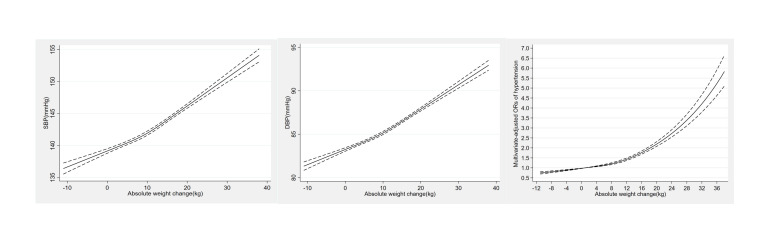
Dose-response association of weight gain from young to middle adulthood with blood pressure and the risk of hypertension. Adjusted for age, gender, ethnicity, Hukou status, marital status, education level, occupation, household income, smoking status, drinking status, leisure time physical activity, family history of hypertension, and comorbidities (diabetes, CVD, COPD, and cancer). The dashed lines represent the 95% CIs.

Regarding the dose-response relationship of absolute weight changes with blood pressure and the risk of hypertension (**Figure 2**), we noted a linearly positive association of absolute weight changes with SBP (*P*-value for linearity <0.001), DBP (*P*-value for linearity <0.001) and a non-linear positive association with hypertension risk (*P*-value for nonlinearity <0.001).

## DISCUSSION

In this population-based study in Shandong Province, China, we found that participants who maintained a stable normal weight from young to middle adulthood had the lowest levels of elevated blood pressure and incidence of hypertension, while those with stable obesity and those who gained weight were associated with increases in blood pressure and hypertension risk. These findings demonstrate that public health strategies should prioritise weight maintenance across the whole life course, beginning in young adulthood, to mitigate the burden of elevated blood pressure and hypertension.

The links between weight change or BMI change and elevated blood pressure or hypertension risk evident in our findings were also observed in research worldwide [30–38]. However, data from low-income settings remained comparatively scarce, with most studies being conducted in Western countries. The Framingham Heart Study [30] found that a 5% increase in weight could elevate the odds of developing hypertension by 20–30%, while an analysis of 9297 participants from a British birth cohort found that high BMI and excess BMI gain at any life stage were associated with increased blood pressure [31]. Research conducted in the USA [34] confirmed a dose-dependent relationship, indicating that higher body weight was associated with progressively increasing hypertension risk. This consistent pattern highlighted weight gain as a key modifiable risk factor across diverse populations. Here, we uniquely observed a J-shaped or U-shaped association between weight change and hypertension risk.

One key finding of our research is the significantly stronger association of persistent obesity with elevated SBP, DBP, and hypertension risk in females compared to males. This gender disparity aligns with studies conducted in the USA, which showed that women maintaining chronic obesity were at a higher risk of hypertension than men [39]. Potential mechanisms include a combination of adipose tissue endocrine dysregulation, overactivation of the renin-angiotensin-aldosterone system, sympathetic nervous system hyperactivity, and reductions in sex hormone levels, particularly after menopause [40].

Achieving sustained weight control proved challenging in our study: only 1.15% of participants transitioned from the obesity range at age 25 to the non-obesity range when they were surveyed. Despite weight reduction, this subgroup exhibited persistently elevated blood pressure, suggesting that sustained weight loss alone may be insufficient to reverse hypertension risk, possibly reflecting irreversible vascular damage from prolonged obesity or weight loss secondary to underlying illness. Critically, obesity at any point from young to middle adulthood was associated with increased blood pressure and hypertension risk, indicating a long-term detrimental effect on blood pressure regulation. Therefore, effective weight management throughout the life cycle is essential. We did not, however, find a significant association between weight loss and blood pressure, possibly because these individuals had a lower BMI at age 25 and because their weight loss mainly consisted of lean, rather than fat mass.

This study is grounded in China's unique socioeconomic trajectory. Some participants born in the 1950–60s experienced scarcity related to the planned economy during their young adulthood (1970–80s), while the subsequent market liberalisation dramatically transformed their diets and occupations. This transition differs distinctly from the modern-day context in China, so extrapolating these weight gain-hypertension associations to young populations today requires caution.

The exact underlying pathophysiological mechanisms by which change in weight is associated with blood pressure remain unclear. Weight gain might play an important role in elevated blood pressure by increasing in total circulating blood volumes and cardiac output, accompanied by increased circulating plasma catecholamine levels [41,42]. The activation of the sympathetic nervous system is another consequence of obesity, while insulin and leptin are also likely to be involved [43]. Stimulating the renin-angiotensin system and physical compression of the kidney may also be important factors in this sense [44].

Several limitations should be acknowledged when interpreting our findings. First, we relied on self-reported weight data from participants at the age of 25 years, which may introduce recall bias. Relatedly, we note that the sensitivity analyses revealed differences in some characteristics between the study sample and the participants lacking recalled weight at age 25 (Table S4 in the **Online Supplementary Document**), supporting this concern. Recall accuracy may likewise vary between subgroups based on current BMI, health status, or age, potentially leading to misclassification. However, prior validation studies and a recent meta-analysis [45] suggested that recalled early life body weight might be an effective method for life-course epidemiological analyses. We likewise note that many large-scale studies have utilised recalled and self-reported weight [22,28]. Second, we only collected data on body weight at two time points, limiting our ability to assess weight cycling during this period. Third, the non-random selection of counties may limit the generalisability of our results beyond Shandong Province and introduce potential selection bias. Fourth, while we calculated changes in weight over time by subtracting historical weight from current weight, the cross-sectional design of our study limits causal inference regarding the associations between weight changes and blood pressure. Fifth, due to the nature of a retrospective database study, important information such as dietary sodium and medication use (which may affect weight change or blood pressure) was unavailable, introducing potential bias. Sixth, ORs may overestimate relative risk given hypertension prevalence >50%; however, consistent results across linear (blood pressure) and logistic (hypertension) models support robustness.

## CONCLUSIONS

In this sample of participants from the Shandong Province, we observed that weight changes throughout young to middle adulthood were associated with elevated blood pressure and an incidence of hypertension. However, potential recall bias, reverse causation, and unmeasured confounders limit causal inference. Future prospective studies with serial weight measurements are needed to confirm these associations. We recommend integrating weight trajectory assessment into routine hypertension screening and electronic health records to enable early and personalised prevention, and call for the adoption of weight management strategies across the life course in public health interventions in order to more effectively reduce the country’s CVD burden.

## Additional material


Online Supplementary Document
