# Supplementary material for: Weight changes from young to middle adulthood in relation to blood pressure and hypertension
Source: J Glob Health. 2025 Oct 24;15:04297. doi: 10.7189/jogh.15.04297 (PMC12548777; doi:10.7189/jogh.15.04297)
Supplement: Online Supplementary Document [file jogh-15-04297-s001.pdf]

**Supplement to: Yu X, Li X, Xu C, Duan X, Liu D, Dong J, Ren J, Tang J, Xu A, Guo X. Weight changes from young to middle adulthood in relation to blood pressure and hypertension. J Glob Health. 2025;15:04297.**

**Table S1.** β or OR and 95% CIs of blood pressure with BMI status

| BMI status† | SBP | | | | DBP | | | | Hypertension | | | |
|---|---|---|---|---|---|---|---|---|---|---|---|---|
| | Model 1* | | Model 2* | | Model 1* | | Model 2* | | Model 1* | | Model 2* | |
| | β(95% CI) | P | β(95% CI) | P | β(95% CI) | P | β(95% CI) | P | OR(95% CI) | P | OR(95% CI) | P |
| BMI at age 25 years | | | | | | | | | | | | |
| Underweight | -1.27(-1.94,-0.59) | <0.001 | -1.88(-2.72,-1.03) | <0.001 | -0.60(-0.97,-0.24) | <0.001 | -1.12(-1.61,-0.64) | <0.001 | 0.85(0.79,0.90) | <0.001 | 0.79(0.72,0.87) | <0.001 |
| Normal weight (ref) | 1.00 | | 1.00 | | 1.00 | | 1.00 | | 1.00 | | 1.00 | |
| Overweight | 3.99(2.57,4.42) | <0.001 | 2.91(2.38,3.44) | <0.001 | 1.29(1.05,1.52) | <0.001 | 1.37(1.07,1.67) | <0.001 | 1.47(1.41,1.53) | <0.001 | 1.37(1.29,1.46) | <0.001 |
| Obesity | 7.54(6.56,8.53) | <0.001 | 6.38(5.18,7.57) | <0.001 | 3.48(2.94,4.02) | <0.001 | 3.06(2.39,3.74) | <0.001 | 2.24(2.02,.49) | <0.001 | 2.14(1.85,2.48) | <0.001 |
| BMI at survey | | | | | | | | | | | | |
| Underweight | -5.64(-7.70,-3.58) | <0.001 | -7.38(-9.89,-4.87) | <0.001 | -3.11(-4.23,-1.99) | <0.001 | -4.10(-5.51,-2.68) | <0.001 | 0.72(0.58,0.89) | 0.003 | 0.63(0.47,0.85) | 0.003 |
| Normal weight (ref) | 1.00 | | 1.00 | | 1.00 | | 1.00 | | 1.00 | | 1.00 | |
| Overweight | 5.92(5.52,6.32) | <0.001 | 5.28(4.79,5.77) | <0.001 | 3.53(3.32,3.75) | <0.001 | 3.32(3.04,3.59) | <0.001 | 1.82(1.75,1.90) | <0.001 | 1.84(1.74,1.94) | <0.001 |
| Obesity | 11.93(11.48,12.38) | <0.001 | 11.39(10.83,11.95) | <0.001 | 6.93(6.69,7.18) | <0.001 | 7.01(6.70,7.33) | <0.001 | 3.43(3.28,3.60) | <0.001 | 3.84(3.59,4.11) | <0.001 |

OR - odd ratios, CI - confidence interval, ref - reference

*Model 1. was unadjusted. Model 2. was adjusted for age, gender, ethnicity, Hukou status, marital status, education level, occupation, household income, smoking status, drinking status, leisure time physical activity, family history of hypertension, and comorbidities.

†underweight (BMI＜18.5 kg/$m^2$), normal weight (18.5 kg/$m^2$≤ BMI <24.0 kg/$m^2$), overweight (24.0 kg/$m^2$≤ BMI <28.0 kg/$m^2$), and obesity (BMI ≥28.0 kg/$m^2$).

**Table S2.** $\beta$ or OR and 95% CIs of blood pressure with weight change from young to middle adulthood

| Weight change group* | SBP | | DBP | | Hypertension | |
|---|---|---|---|---|---|---|
| | **$\beta$ (95% CI)** | **P** | **$\beta$ (95% CI)** | **P** | **OR (95% CI)** | **P** |
| Weight loss group | 0.52(-0.39,1.43) | 0.264 | -0.51(-1.03,0.01) | 0.050 | 1.01(0.91,1.11) | 0.908 |
| Stable weight group (ref) | 1.00 | | 1.00 | | 1.00 | |
| Small to moderate weight gain | 2.03(1.35,2.70) | <0.001 | 1.18(0.80,1.56) | <0.001 | 1.20(1.11,1.30) | <0.001 |
| Moderate to large weight gain | 5.39(4.72,6.07) | <0.001 | 3.24(2.86,3.62) | <0.001 | 1.75(1.62,1.88) | <0.001 |
| Extreme weight gain group | 9.95(9.15,10.74) | <0.001 | 6.21(5.77,6.66) | <0.001 | 3.00(2.73,3.28) | <0.001 |

OR - odd ratios, CI - confidence interval, ref - reference

*weight loss group (weight loss≥2.5 kg), stable weight group (weight change within 2.5 kg), small to moderate weight gain (2.5 kg≤ weight gain <10.0 kg), moderate to large weight gain (10 kg≤ weight gain <20.0 kg), and extreme weight gain group (weight gain ≥20.0 kg).

Model was adjusted for age, gender, ethnicity, Hukou status, marital status, education level, occupation, household income, smoking status, drinking status, leisure time physical activity, family history of hypertension, and comorbidities.

**Table S3.** *β* or OR and 95% CIs of blood pressure with weight change from young to middle adulthood, stratified by gender and age

| Weight change group* | SBP | | DBP | | Hypertension | |
|---|---|---|---|---|---|---|
| | ***β* (95% CI)** | **P** | ***β* (95% CI)** | **P** | **OR (95% CI)** | **P** |
| Male | | | | | | |
| Weight loss group | 1.29(-0.16,2.74) | 0.081 | -0.14(-0.98,0.70) | 0.745 | 1.06(0.90,1.25) | 0.459 |
| Stable weight group (ref) | 1.00 | | 1.00 | | 1.00 | |
| Small to moderate weight gain | 2.25(1.13,3.37) | <0.001 | 0.93(0.28,1.58) | 0.005 | 1.21(1.06,1.37) | 0.004 |
| Moderate to large weight gain | 5.43(4.32,6.54) | <0.001 | 3.25(2.61,3.89) | <0.001 | 1.86(1.64,2.11) | <0.001 |
| Extreme weight gain group | 9.27(8.05,10.50) | <0.001 | 5.95(5.24,6.65) | <0.001 | 3.00(2.60,3.46) | <0.001 |
| Female | | | | | | |
| Weight loss group | 0.03(-1.13,1.20) | 0.954 | -0.77(-1.42,-0.13) | 0.019 | 0.97(0.85,1.10) | 0.627 |
| Stable weight group (ref) | 1.00 | | 1.00 | | 1.00 | |
| Small to moderate weight gain | 1.89(1.05,2.73) | <0.001 | 1.30(0.84,1.77) | <0.001 | 1.20(1.09,1.32) | <0.001 |
| Moderate to large weight gain | 5.17(4.32,6.02) | <0.001 | 3.17(2.69,3.64) | <0.001 | 1.65(1.50,1.82) | <0.001 |
| Extreme weight gain group | 10.12(9.07,11.18) | <0.001 | 6.28(5.69,6.86) | <0.001 | 2.87(2.54,3.24) | <0.001 |
| Age 35-49 years | | | | | | |
| Weight loss group | 1.25(-0.57,3.07) | 0.177 | -0.01(-1.14,1.11) | 0.983 | 1.27(1.01,1.64) | 0.042 |
| Stable weight group (ref) | 1.00 | | 1.00 | | 1.00 | |
| Small to moderate weight gain | 2.23(1.06,3.40) | <0.001 | 0.84(0.11,1.56) | 0.024 | 1.20(1.03,1.42) | 0.023 |
| Moderate to large weight gain | 5.92(4.76,7.09) | <0.001 | 3.00(2.28,3.72) | <0.001 | 1.87(1.59,2.19) | <0.001 |
| Extreme weight gain group | 11.40(10.06,12.74) | <0.001 | 6.51(5.68,7.34) | <0.001 | 3.44(2.89,4.11) | <0.001 |
| Age 50-64 years | | | | | | |
| Weight loss group | 0.25(-0.80,1.31) | 0.637 | -0.58(-1.16,-0.04) | 0.048 | 0.97(1.00,1.62) | 0.053 |
| Stable weight group (ref) | 1.00 | | 1.00 | | 1.00 | |
| Small to moderate weight gain | 1.90(1.09,2.72) | <0.001 | 1.23(0.79,1.67) | <0.001 | 1.21(1.02,1.41) | 0.032 |
| Moderate to large weight gain | 5.05(4.23,5.87) | <0.001 | 3.18(2.73,3.62) | <0.001 | 1.83(1.56,2.15) | <0.001 |
| Extreme weight gain group | 9.06(8.09,10.03) | <0.001 | 5.85(5.32,6.37) | <0.001 | 3.36(2.82,4.02) | <0.001 |

OR - odd ratios, CI - confidence interval, ref - reference

*weight loss group (weight loss≥2.5 kg), stable weight group (weight change within 2.5 kg), small to moderate weight gain (2.5 kg≤ weight gain <10.0 kg), moderate to large weight gain (10 kg≤ weight gain <20.0 kg), and extreme weight gain group (weight gain ≥20.0 kg).

Model was adjusted for age, gender, ethnicity, Hukou status, marital status, education level, occupation, household income, smoking status, drinking status, leisure time physical activity, family history of hypertension, and comorbidities.

**Table S4.** Descriptive characteristics of the study sample and participants without recalled weight at age 25

| Characteristics | Study sample (n=56,459) | participants without recalled weight at age 25 (n=11,402) | *P* |
|---|---|---|---|
| Age (mean±SD , years) | 53.34±7.38 | 53.38±7.42 | 0.525 |
| Gender | | | |
| Male | 21,996(38.96) | 4,517(39.62) | 0.190 |
| Female | 34,463(61.04) | 6,885(60.38) | |
| Ethnicity | | | **＜0.001** |
| Han | 56,311(99.74) | 11,347(99.52) | |
| Non-Han | 148(0.26) | 55(0.48) | |
| Hukou status | | | **＜0.001** |
| Urban | 48,706(86.27) | 10,252(89.91) | |
| Rural | 3,780(6.70) | 730(6.40) | |
| Unified residential status | 3,971(7.03) | 420(3.68) | |
| Unknown* | 2(0.00) | / | |
| Marital status | | | **＜0.001** |
| Married | 54,024(95.69) | 10,772(94.47) | |
| Unmarried | 2,435(4.31) | 630(5.53) | |
| Education level | | | **＜0.001** |
| Primary school or lower | 26,338(46.65) | 4,859(49.95) | |
| Middle school | 22,008(38.98) | 3,518(36.16) | |
| High school | 6,425(11.38) | 926(9.52) | |
| College or above | 1,291(2.29) | 176(1.81) | |
| Unknown* | 397(0.70) | 249(2.56) | |
| Occupation | | | **＜0.001** |
| Farmer | 44,153(78.20) | 8,548(74.97) | |
| Nonfarmer | 11,923(21.12) | 2,711(23.78) | |
| Unknown* | 383(0.68) | 143(1.25) | |
| Household income(¥/year) | | | **＜0.001** |
| <10,000 | 10,724(18.99) | 2,855(25.04) | |
| 10,000–50,000 | 37,245(65.97) | 6,766(59.34) | |
| >50,000 | 6,452(11.43) | 534(4.68) | |
| Unknown* | 2,038(3.61) | 1,247(10.94) | |
| Smoking status | 10,040(17.78) | 2,120(18.59) | **0.040** |
| Drinking status | 8,133(14.41) | 1,711(15.01) | 0.096 |
| Leisure time physical activity | | | **＜0.001** |
| Never | 41,058(72.72) | 9,673(84.84) | |
| 1 - 3 times a month | 1,706(3.02) | 316(2.77) | |
| 1-2 times a week | 2,667(4.72) | 375(3.29) | |
| 3-5 times a week | 3,344(5.92) | 224(1.96) | |

| | | | |
|---|---|---|---|
| Everyday | 7,647(13.54) | 584(5.12) | |
| Unknown* | 37(0.07) | 230(2.02) | |
| Family history of hypertension | 2,832(5.02) | 112(0.98) | **＜0.001** |
| Comorbidities | | | |
| Diabetes | 4,189(7.42) | 2(7.14) | 0.955 |
| CVD | 2,058(3.65) | 1(3.57) | 0.983 |
| COPD | 61(0.17) | 0(0.00) | 0.827 |
| Cancer | 100(0.31) | 0(0.00) | 0.768 |
| BMI (mean±SD, kg/$m^2$) at survey | 25.97±3.58 | 26.04±3.53 | 0.054 |
| Blood pressure (mmHg) | | | |
| SBP | 143.28±20.85 | 144.43±20.64 | **＜0.001** |
| DBP | 85.01±11.38 | 84.98±11.06 | 0.832 |
| Hypertension | 31,223(55.30) | 6,453(56.60) | **0.011** |

SD - Standard deviation, CVD - Cardiovascular diseases, COPD - chronic obstructive pulmonary disease, BMI - Body mass index, SBP - Systolic blood pressure, DBP - , Diastolic blood pressure.

Student’s T-tests for quantitative variable; Chi-square test for qualitative variable.

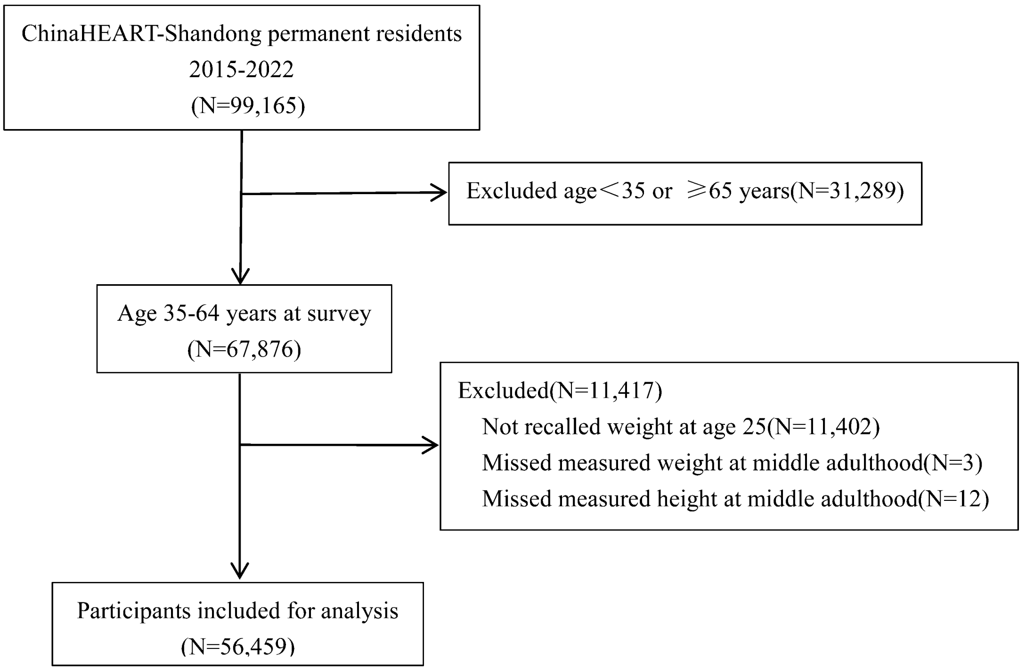


**Figure S1.** Study participant flow
